# HIV-1 Nef Induces Hck/Lyn-Dependent Expansion of Myeloid-Derived Suppressor Cells Associated with Elevated Interleukin-17/G-CSF Levels

**DOI:** 10.1128/JVI.00471-21

**Published:** 2021-08-10

**Authors:** Elena Priceputu, Marc Cool, Nathalie Bouchard, Julio Roberto Caceres-Cortes, Clifford A. Lowell, Zaher Hanna, Paul Jolicoeur

**Affiliations:** a Laboratory of Molecular Biology, Clinical Research Institute of Montreal, Montreal, Quebec, Canada; b Department of Laboratory Medicine, University of California, San Francisco, California, USA; c Department of Medicine, University of Montreal, Montreal, Quebec, Canada; d Microbiology and Immunology, University of Montreal, Montreal, Quebec, Canada; e Division of Experimental Medicine, McGill University, Montreal, Quebec, Canada; Emory University

**Keywords:** HIV, Nef, Hck, Lyn, Src, iNOS, IL-17, G-CSF, HIV, myeloid cells

## Abstract

Human immunodeficiency virus (HIV) or simian immunodeficiency virus (SIV) infection causes myelodysplasia, anemia, and accumulation of inflammatory monocytes (CD14^+^ CD16^+^) through largely unknown cellular and molecular pathways. The mouse cells thought to be equivalent to human CD14^+^ CD16^+^ cells are CD11b^+^ Gr1^+^ myeloid-derived suppressor cells (MDSC). We used HIV transgenic (Tg) mouse models to study MDSC, namely, CD4C/Nef Tg mice expressing *nef* in dendritic cells (DC), pDC, CD4^+^ T, and other mature and immature myeloid cells and CD11c/Nef Tg mice with a more restricted expression, mainly in DC and pDC. Both Tg strains showed expansion of granulocytic and CD11b^+^ Gr1^low/int^ cells with MDSC characteristics. Fetal liver cell transplantation revealed that this expansion was stroma-independent and abrogated in mixed Tg/non-Tg 50% chimera. Tg bone marrow (BM) erythroid progenitors were decreased and myeloid precursors increased, suggesting an aberrant differentiation likely driving CD11b^+^ Gr1^+^ cell expansion, apparently cell autonomously in CD4C/Nef Tg mice and likely through a bystander effect in CD11c/Nef Tg mice. Hck was activated in Tg spleen, and Nef-mediated CD11b^+^ Gr1^+^ cell expansion was abrogated in Hck/Lyn-deficient Nef Tg mice, indicating a requirement of Hck/Lyn for this Nef function. IL-17 and granulocyte colony-stimulating factor (G-CSF) were elevated in Nef Tg mice. Increased G-CSF levels were normalized in Tg mice treated with anti-IL-17 antibodies. Therefore, Nef expression in myeloid precursors causes severe BM failure, apparently cell autonomously. More cell-restricted expression of Nef in DC and pDC appears sufficient to induce BM differentiation impairment, granulopoiesis, and expansion of MDSC at the expense of erythroid maturation, with IL-17→G-CSF as one likely bystander contributor.

**IMPORTANCE** HIV-1 and SIV infection often lead to myelodysplasia, anemia, and accumulation of inflammatory monocytes (CD14^+^ CD16^+^), with the latter likely involved in neuroAIDS. We found that some transgenic (Tg) mouse models of AIDS also develop accumulation of mature and immature cells of the granulocytic lineage, decreased erythroid precursors, and expansion of MDSC (equivalent to human CD14^+^ CD16^+^ cells). We identified Nef as being responsible for these phenotypes, and its expression in mouse DC appears sufficient for their development through a bystander mechanism. Nef expression in myeloid progenitors may also favor myeloid cell expansion, likely in a cell-autonomous way. Hck/Lyn is required for the Nef-mediated accumulation of myeloid cells. Finally, we identified G-CSF under the control of IL-17 as one bystander mediator of MDSC expansion. Our findings provide a framework to determine whether the Nef>Hck/Lyn>IL-17>G-CSF pathway is involved in human AIDS and whether it represents a valid therapeutic target.

## INTRODUCTION

The impact of human immunodeficiency virus type 1 (HIV-1) or simian immunodeficiency virus (SIV) infection on macrophages and dendritic cells (DC) has been relatively well studied (1, 2). However, significantly less is known about the effects of these viruses on monocytes. Human CD14^+^ CD16^+^ monocytes are considered equivalent to mouse inflammatory CD11b^+^ Gr1^+^ monocytes (3). The CD11b^+^ Gr1^+^ cells were first reported to accumulate in cancer-bearing mice and to favor tumor growth (4). Because they are immunosuppressive, they were designated myeloid-derived suppressor cells (MDSC) (4). MDSC are immature, heterogeneous (CD11b^+^ Gr1^low^, CD11b^+^ Gr1^int^, and CD11b^+^ Gr1^high^) (5), and likely originate from myeloid granulocytic/macrophage progenitors (GMP) (4). Indeed, conditions favoring their expansion are associated with accumulation of myeloid precursors, as documented in mice deficient for SHIP (6) or Lyn/Hck (7) genes.

HIV and SIV infections lead to the accumulation of circulating CD14^+^ CD16^+^ monocytes, especially at late stages of disease (8–12). Moreover, circulating CD16^+^ monocytes appear to be the precursors of the CD163^+^ perivascular macrophages infiltrating the central nervous system (CNS) in neuroAIDS (13). The cellular basis, the molecular pathways, and the viral gene responsible for this myeloid cell accumulation remain largely unknown. The availability of a relevant animal model, amenable to easy experimentation with hematopoietic cells, would greatly facilitate the understanding of this virus-induced phenomenon.

In our previous work, we used mouse models of AIDS to study HIV-1 pathogenesis, in particular the CD4C/Nef (previously known as CD4C/HIV-MutG [14]) Tg mice. These Tg mice express only Nef with the regulatory elements of the human CD4 gene (CD4C) in the same cells as those infected with HIV in human, i.e., in CD4^+^ T cells and myeloid cells, and develop an AIDS-like disease (14). They also show abnormalities within two distinct Tg myeloid cell subsets: an enhanced production of ferritin by macrophages (15) and an accumulation of immature CD11c^+^ CD11b^hi^ DC in spleen (16). In addition, in another mouse model, CD11c/Nef Tg mice expressing HIV Nef mainly in myeloid CD11c-positive DC through the regulatory elements of the mouse CD11c gene, we also found accumulation of CD11c^+^ CD11b^hi^ DC in spleen (17). These results suggest that myeloid cell differentiation is affected by Nef in these Tg mouse models.

In the present study, we report that Nef, expressed in these Tg mice, also induces the accumulation of other cell subsets, the CD11b^+^ Gr1^+^ cells, some belonging to the granulocytic lineage and others showing MDSC characteristics.

## RESULTS

### Accumulation of mature and immature myeloid CD11b^+^ Gr1^+^ cells in CD11c/Nef Tg mice associated with increased proliferation and decreased apoptosis.

**FIG 1:**
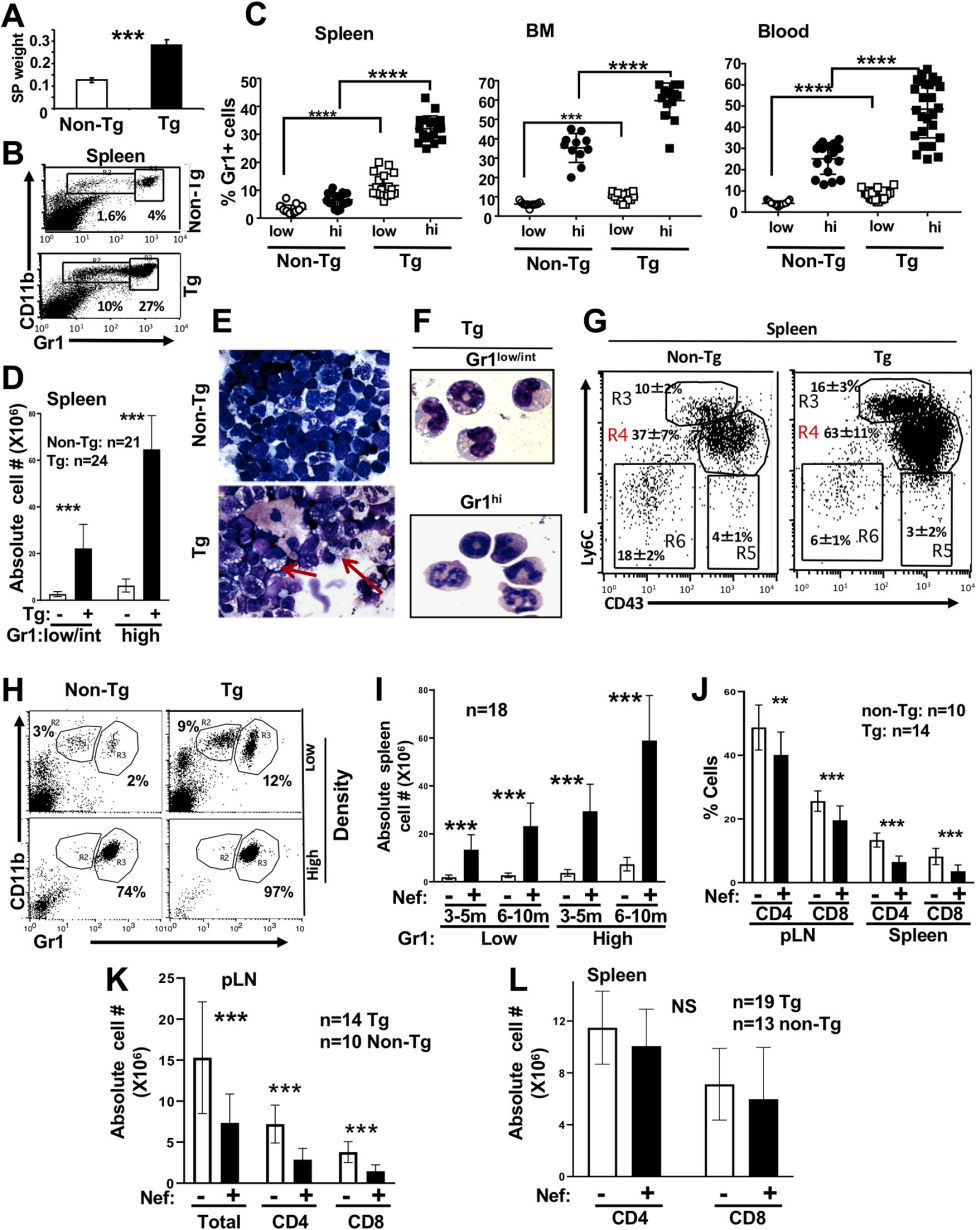
Accumulation of immature myeloid CD11b^+^ Gr1^+^ cells in CD11c/Nef Tg mice. (A) Increase of spleen (SP) weight (g) measured at 10 months of age. Non-Tg (*n* = 6) and Tg (*n* = 6) mice were used. (B to D) Representative FACS profiles (B) and quantification of percentages (C) or absolute cell numbers (D) of CD11c-negative CD11b^+^ Gr1^low/int^ and CD11b^+^ Gr1^hi^ cells in spleen, BM, and blood of 4- to 6-month-old Tg (+) and control non-Tg (−) mice. (E and F) Wright-Giemsa staining of total spleen cells from Tg and non-Tg mice, total spleen cells (E) and Tg splenocyte Gr1^low/int^ or Gr1^hi^ subsets purified by cell sorting (F). Note abundant vacuoles in Tg cells and different morphology of Gr1^low/int^ and Gr1^hi^ cells. (G) Representative FACS profiles and quantification of percentage of Ly6C^++^ CD43^++^ spleen cells of non-Tg (*n* = 5) and Tg (*n* = 5) mice stained for Ly6C and CD43 gated on CD11b^+^ cells. The percentage of Ly6C^+^ CD43^++^ (R4) cells was significantly different (*P* ≤ 0.01) in Tg versus non-Tg mice, but the two other Ly6C/CD43 cell subsets (R3 and R5) were not significantly different. Statistical analysis was performed using Student’s *t* test. (H) Blood of Tg and non-Tg mice was separated on a Ficoll gradient, as described in Materials and Methods, and cells were collected in the high- or low-density region of the tube and stained for CD11b and Gr1. A representative FACS profile is shown. (I) The number of spleen CD11b^+^ Gr1^low/int^ and CD11b^+^ GR1^hi^ cells was measured in CD11c/Nef Tg (+) and non-Tg (−) mice of different ages. Data were obtained from FACS analysis. (J to L) T cells in spleen and peripheral LN (pLN) of CD11c/Nef Tg (+) and non-Tg (−) mice were assessed by FACS analysis after staining for CD4 and CD8 and expressed as percent (J) or absolute cell number (K and L). Note that the percentage of both CD4^+^ and CD8^+^ T cells tends to be decreased in both organs (J), but the absolute number of both T cell subsets is decreased in LN (K) but not in spleen (L). **, *P* ≤ 0.01; ***, *P* ≤ 0.001.

**FIG 2:**
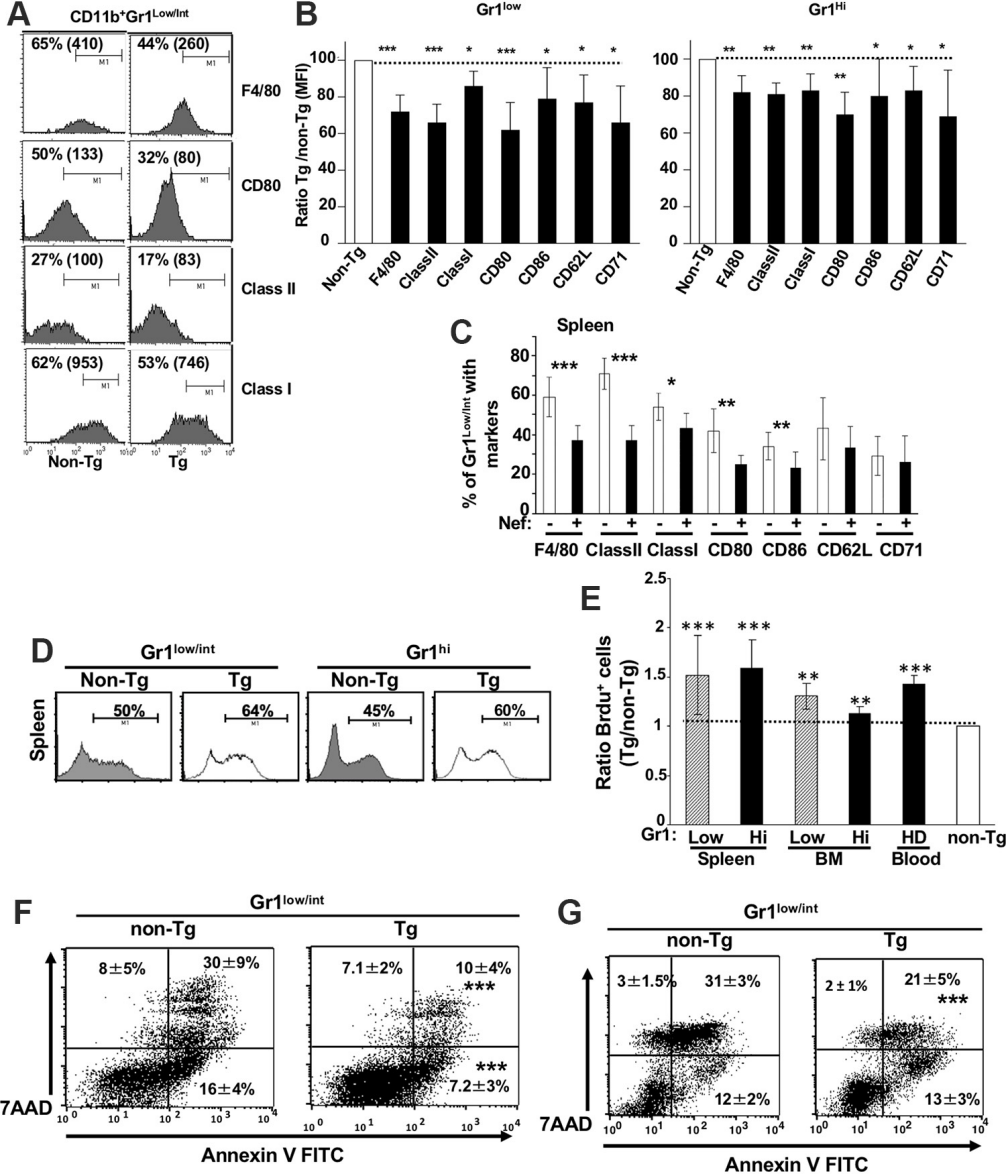
Analysis of maturation/activation markers, proliferation, and death/apoptosis of CD11b^+^ Gr1^+^ cell subsets from CD11c/Nef Tg mice. (A to C) Representative FACS profiles (A) and mean fluorescence intensity (MFI) (B) of the indicated cell surface markers on CD11b^+^ Gr1^low^ and CD11b^+^ Gr1^hi^ spleen cells from Tg (*n* = 15) and non-Tg (*n* = 15) mice and quantification (C) of the percentage spleen CD11b^+^ GR1^low^ cells from Tg (+) and non-Tg (−) mice expressing the indicated cell surface markers. (D and E) Representative FACS profiles (D) and calculated ratios (Tg/non-Tg) of percentage (E) of purified BrdU-positive CD11c-negative CD11b^+^ Gr1^+^ cells in spleen, BM, and blood of Tg and non-Tg mice. Blood HD cells are from a high-density Ficoll gradient. A value of 1 was assigned to non-Tg cells. Results are from three independent experiments. (F and G) Representative FACS profiles of apoptotic/dead cells and quantification of percentage of spleen CD11b^+^ Gr1^low/int^ cell subsets *ex vivo* (F) or of purified CD11b^+^ Gr1^low^ spleen cells cultured *in vitro* for 24 h in the presence of LPS (G). Cells were stained for cell surface CD11c, CD11b, Gr1, 7-AAD, and annexin V and gated on CD11c-negative cells. Statistical analysis was performed using Student’s *t* test (*, *P* ≤ 0.05; **, *P* ≤ 0.01; ***, *P* ≤ 0.001).

CD11c/Nef Tg mice show splenomegaly (Fig. 1A). Total spleen cells for Tg mice (*n* = 24) was 196 × 10^6^ ± 36 × 10^6^ compared to 85 × 10^6^ ± 14 × 10^6^ for non-Tg mice (*n* = 21) (***, *P* ≤ 0.001). Accumulation of immature DC, CD11b^+^ F4/80^+^ (17), CD11b^+^ Gr1^hi^ mature neutrophils, and CD11b^+^ Gr1^low/int^ immature cells was observed mainly in spleen, blood, and BM (Fig. 1B to D). Morphologically, myeloid cells show more abundant and larger vacuoles than non-Tg ones (Fig. 1E and F). CD11b^+^ Gr1^hi^ cells have heterogeneous segmented nuclei, as neutrophils, whereas CD11b^+^ Gr1^low/int^ cells showed doughnut-like nuclei (Fig. 1F) found in less mature myeloid cells. Labeling of CD11b^+^ cells with CD43 and Ly6C revealed increased percentages of CD43^++^ Ly6C^++^ intermediate inflammatory monocytes in blood (data not shown) and spleen (Fig. 1G) of Tg mice. The high-density region of Ficoll gradient contains mainly CD11b^+^ Gr1^hi^ neutrophils, as expected, and these accumulate in Tg mice, whereas the low-density region contains Gr1^low^ and Gr1^hi^ cells, both also in higher proportion in Tg mice (Fig. 1H). This myeloid cell accumulation progresses with age (Fig. 1I) and is associated with low body weight (data not shown) and a lower proportion of CD4^+^ and CD8^+^ T cells (Fig. 1J to L). In addition to morphology, the lower mean fluorescence intensity and percentages of F4/80, CD80, CD86, major histocompatibility complex class I (MHC-I), and MHC-II (Fig. 2A to C) also suggest an immature phenotype of these accumulating CD11b^+^ Gr1^+^ cells.

To determine whether accumulation of these myeloid cell populations was caused by enhanced proliferation, mice were fed with bromodeoxyuridine (BrdU). Incorporation of BrdU was increased in Tg CD11b^+^ Gr1^+^ cells from spleen, blood, and BM relative to non-Tg cells (Fig. 2D and E). We also evaluated survival of these cells. Both subsets of Tg CD11b^+^ Gr1^+^ (Gr1^low/int^ or Gr^1/hi^ [data not shown]) either among total spleen cells *ex vivo* (Fig. 2F) or purified and cultured in the presence of apoptotic stimuli (LPS or GM-CSF [data not shown]) for 24 h (Fig. 2G) showed increased survival relative to non-Tg ones, as assessed by 7-aminoactinomycin D (7-AAD) and annexin V staining. These data suggest that accumulation of Tg CD11b^+^ Gr1^+^ cells results from their increased proliferation and resistance to cell apoptosis/death.

### Inducible nitric oxide synthase (iNOS)-mediated T cell suppressive activity of CD11b^+^ Gr1^low/int^ cells from CD11c/Nef Tg mice.

**FIG 3:**
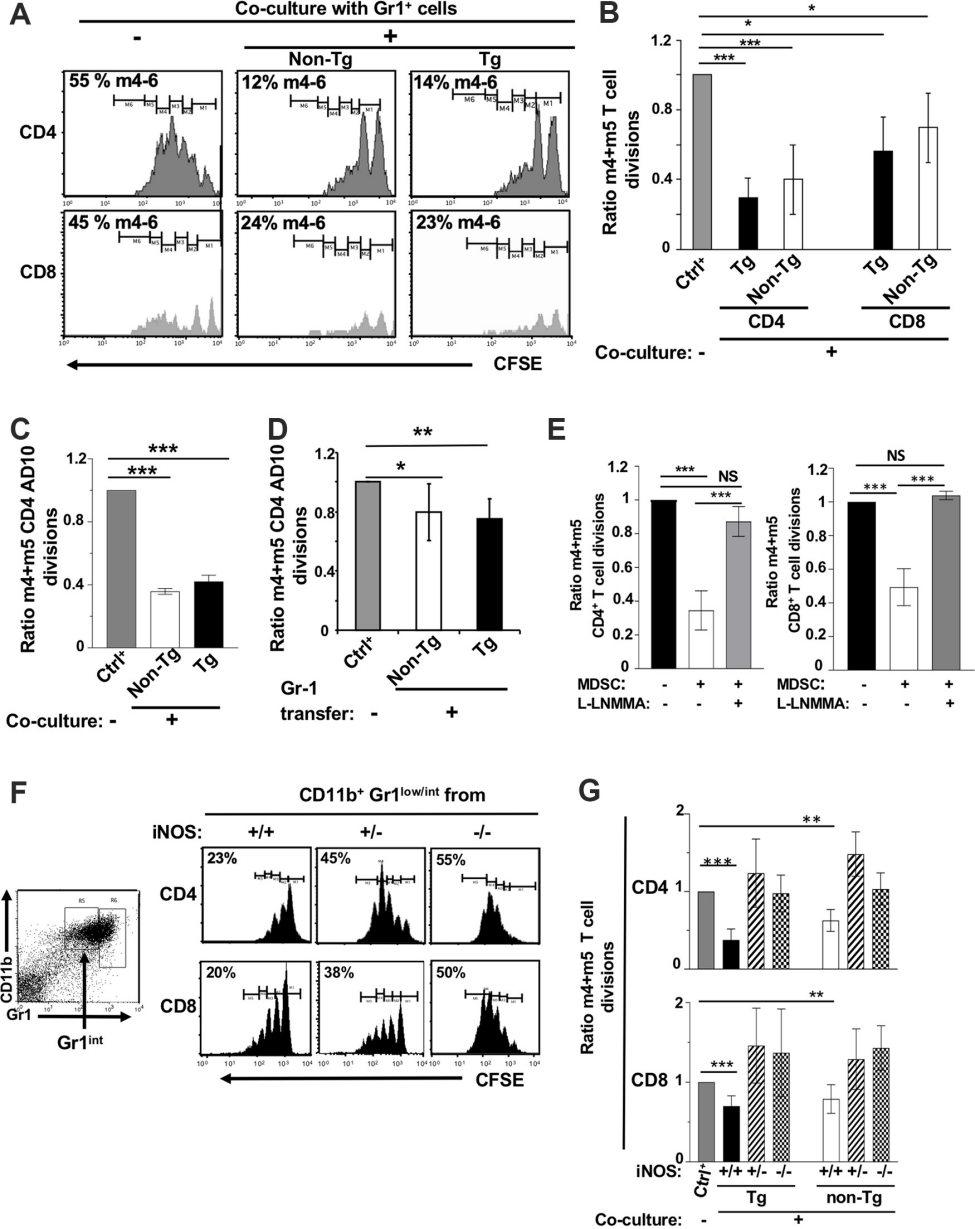
CD11b^+^ GR1^low/int^ cells from CD11c/Nef Tg mice show T cell suppressive activity mediated by iNOS. Sorted purified CD11b^+^ Gr1^+^ cells (10^5^) from Tg or non-Tg mice were cocultured for 3 days with CFSE-labeled normal CD4^+^ or CD8^+^ T cells (10^5^) purified by cell sorting and stimulated with anti-CD3 and anti-CD28 to induce their proliferation. (A and B) Representative FACS profiles (A) and quantification (B) of CFSE dilution in non-Tg CD4^+^ and CD8^+^ T cells cocultured (+) or not (−) with purified CD11b^+^ Gr1^low/int^ cells from Tg or non-Tg mice. In panel B, data are expressed as a ratio of the percentage of stimulated T cells reaching 4 and 5 divisions (m4 + m5) in the presence (+) of CD11b^+^ Gr1^low/int^ cells over those cultured in their absence (−) (normalized at 1). Results from nine experiments are shown. (C) CFSE dilution of purified CD4^+^ T cells from AD10 TcR Tg mice stimulated with Pcc and cocultured (+) or not (−) with CD11b^+^ Gr1^low/int^ cells from non-Tg or Tg mice. Values are from three experiments and expressed as in panel B. (D) *In vivo* adoptive transfer. CFSE-labeled CD4^+^ T cells (3 × 10^6^ to 4 × 10^6^) from AD10 TcR Tg mice were transferred by i.v. tail vein inoculation into normal recipient B10BR mice; 48 h later, mice were injected i.v. with non-Tg or Tg purified CD11b^+^ Gr1^low/int^ cells (2 × 10^6^ to 3× 10^6^) and, within 1 h, inoculated s.c. with Pcc-loaded DC (3 × 10^6^). Values normalized at 1 in the absence of transfer are from 3 independent experiments (*n* = 5 Tg and 5 non-Tg). (E) Quantification of CFSE dilutions of non-Tg CD4^+^ (left) or CD8^+^ (right) T cells cocultured with Tg CD11b^+^ Gr1^low/int^ cells, as described for panel B, in the presence (+) or not (−) of l-NMMA. Values are from 3 independent experiments. (F and G) Representative FACS profile (F) and quantification (G) of CFSE dilution of purified non-Tg CD4^+^ and CD8^+^ T cells cocultured with purified CD11b^+^ Gr1^low/int^ cells from wild-type (+/+) or iNOS-deficient heterozygous (+/−) or homozygous (−/−) CD11c/Nef Tg mice. Values are expressed as in panel B. Data are from five experiments. *, *P* ≤ 0.05; **, *P* ≤ 0.01; ***, *P* ≤ 0.001.

CD11b^+^ Gr1^low/int^ cells have been shown to have suppressive activity (4) against CD4^+^ and CD8^+^ T cells in models of mouse tumors or in other contexts (4). To determine whether Tg CD11b^+^ Gr1^+^ cells exhibit T cell-suppressive functions, purified CD11b^+^ Gr1^+^ cells were cocultured with sorted-purified CFSE-labeled, normal C3H (Fig. 3A and B) or AD10 TcR Tg (Fig. 3C) T cells stimulated *in vitro* with anti-CD31 and CD28 or Pcc antigen, respectively. AD10 TcR mice were used to facilitate readout, as all their T cells harbor a single TcR recognizing a single antigen (Pcc). After 3 days in culture, CFSE fluorescence (reflecting the number of cell divisions) was analyzed by a fluorescence-activated cell sorter (FACS). In this assay, both Tg and non-Tg CD11b^+^ GR1^low/int^ cells suppressed CD4^+^ and CD8^+^ T cell division to the same extent compared to division of T cells in the absence of suppressor cells (Fig. 3A to C), indicating that Tg cells have retained, but not increased, suppressive activities on a per-cell basis. In contrast, in the same assay, Tg or non-Tg CD11b^+^ Gr1^hi^ cells showed no suppressive activity (data not shown), as expected.

We next measured the *in vivo* suppressive functions of Tg and non-Tg CD11b^+^ Gr1^low/int^ cells by their adoptive transfer into normal B10BR mice, along with CSFE-labeled Tg AD10 TcR CD4^+^ T cells stimulated with their cognate antigen (Pcc) presented by DC. FACS analysis was performed by gating on AD10 TcR^+^ CFSE^+^ CD4^+^ T cells. This analysis showed that both Tg and non-Tg CD11b^+^ Gr1^low/int^ cells had the ability to modestly suppress proliferation of AD10 CD4^+^ T cells *in vivo* (Fig. 3D). The lower levels of suppression observed *in vivo*, compared to *in vitro*, may reflect the purity of CD11b^+^ Gr1^low/int^ cell preparations. Because the CD11b^+^ Gr1^+^ purification technique used for *in vitro* suppression experiments (cell sorting) was inadequate to obtain the high number of cells required for *in vivo* suppression in a sufficient number of mice, we had to use an alternative purification strategy (MACS anti-biotin beads). This led to preparations of CD11b^+^ Gr1^low/int^ cells of lower purity (70% versus 95%). Our use of target CD4^+^ T cells for *in vivo* experiments also may have contributed to lower suppression. It was reported that the same MDSC can suppress proliferation of CD4^+^ T cells *in vivo* less efficiently than that of CD8^+^ T cells (18). Finally, it is possible that Pcc-driven signaling for AD10 CD4^+^ T cell proliferation inherently is less responsive to MDSC suppression *in vivo*. We already documented that the percentage of CD4^+^ T cells stimulated by Pcc (m4 + m5 CFSE divisions), in the absence of CD11b^+^ Gr1^+^ cells (control group), was substantially higher *in vivo* than *in vitro* (80% ± 3% versus 61% ± 7%).

NO production is one of the main pathways of suppression by monocytic CD11b^+^ Gr1^low^ cells (4). We first assessed the involvement of iNOS in suppression activity by coculturing non-Tg CD4^+^ or CD8^+^ T cells with purified suppressive CD11b^+^ Gr1^low/int^ cells in the presence or not of l-NMMA, an iNOS inhibitor. l-NMMA restored >90% of T cell division in such cocultures (Fig. 3E), strongly suggesting that NO is involved in the suppressive functions of Tg CD11b^+^ Gr1^low/int^ cells.

To further evaluate the contribution of NO in suppressive activities of CD11b^+^ Gr1^low/int^ cells, we next bred the CD11c/Nef Tg mice on the iNOS gene-deficient background and assayed the *in vitro* T cell-suppressive activity of CD11b^+^ Gr1^low/int^ cells from these mice. CD11b^+^ Gr1^low/int^ spleen cells from heterozygous (+/−) and homozygous (−/−) iNOS-deficient non-Tg or Nef Tg mice had decreased suppressive activity against CD4^+^ and CD8^+^ T cell proliferation relative to control CD11b^+^ Gr1^low/int^ cells from iNOS wild-type (WT; +/+) non-Tg or Nef Tg mice (Fig. 3F and G). These results suggest that Tg CD11b^+^ Gr1^low/int^ cells suppress T cell proliferation largely through the iNOS pathway.

Together, these data show that Tg CD11b^+^ Gr1^low/int^ cells have retained NO as a major mechanism of suppression, similar to that found in normal non-Tg cells, on a per-cell basis. Thus, CD11b^+^ Gr1^low/int^ cells, which accumulate in Nef Tg mice and have suppressive activities, appear to be MDSC.

### Accumulation of early myeloid progenitors in CD11c/Nef Tg mice.

To explore the possible origin of MDSC and other cells of the granulocytic lineage, we next analyzed hematopoietic progenitors. Multipotent BM hematopoietic cells have been defined as the lin^−^ Sca1^+^ c-kit^+^ (LSK) population. For myeloid cell production, LSK differentiate and produce common myeloid progenitors (CMP). CMP differentiate further to give rise to either granulocyte-macrophage progenitors (GMP), megakaryocyte-erythrocyte progenitors (MEP), or monocyte-macrophage-DC progenitors (MDP). GMP can differentiate into both granulocytes (neutrophils) and monocytes (19). In addition, both GMP and MDP can yield LyC6^hi^ and LyC6^−^ CD43^+^ monocytes (20), whereas MDP is also the precursor of CD11c^+^ DC (21).

**FIG 4:**
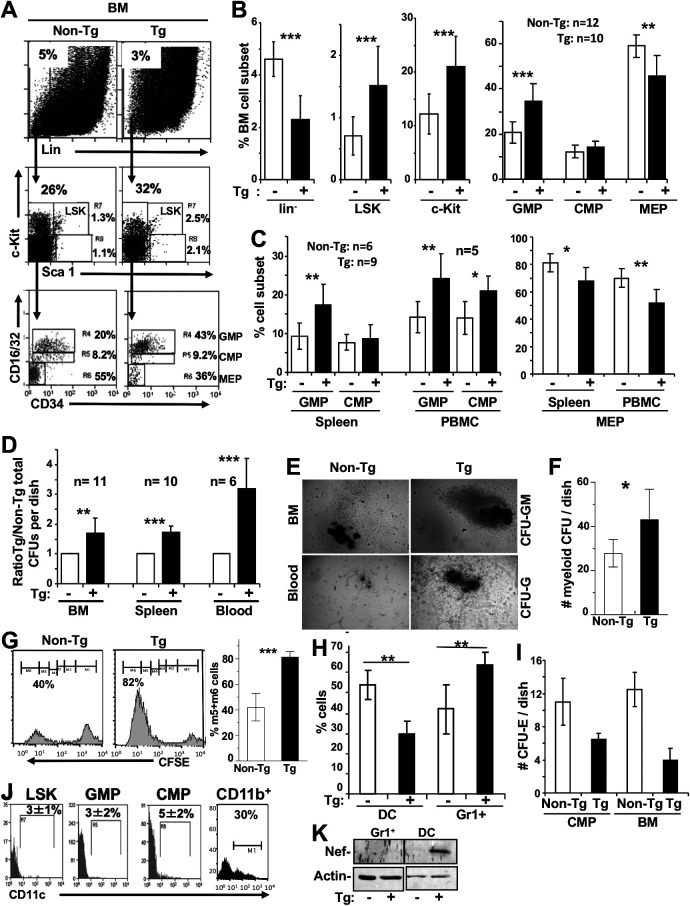
Accumulation of early myeloid progenitors in CD11c/Nef Tg mice. (A to C) Representative FACS profiles of lin^−^ BM cells (A) or percentage of the indicated myeloid progenitors in BM (B), spleen, and PBMC (C) of Tg (+) and non-Tg (−) mice. (D) Colony assays for myeloid progenitors. The same numbers of cells from blood (10^5^), spleen (5 × 10^4^), and BM (10^3^) of Tg and non-Tg mice were plated in methylcellulose as described in Materials and Methods, and colonies were counted 10 days later. (E) Morphology of representative colonies from blood (12 days/culture) and BM (8 days/culture) of Tg and non-Tg mice. Note the larger size of Tg colonies. (F) Quantification of BM CFU-GM/-M/-G colonies per petri dish (*n* = 6). Results are from three experiments. (G) Proliferation of colonies *in vitro*. Methylcellulose colonies were generated from BM of Tg and non-Tg cells, and CFU-GM colonies were picked, labeled with CFSE, and replated at the same number (10^5^). Colonies were harvested after 4 to 7 days and analyzed by FACS to assess CFSE dilution (left), which was quantitated (right). Results are from two experiments. (H) Percentage of DC (CD11b^+^ CD11c^+^) and CD11b^+^ Gr1^+^ cells generated by BM cells of Tg (+) and non-Tg (−) mice incubated in the presence of IL-4 + GM-CSF. Results are from 3 experiments. (I) *In vitro* generation of erythroid colonies. Purified cell-sorted CMP (10^3^) or total BM (2 × 10^4^) cells from non-Tg and Tg mice were cultured in methylcellulose supplemented with Epo alone, and erythroid colonies were counted 5 days later. Results are from two experiments. Note the decreased number of colonies in Tg mice. (J) Expression of CD11c. Representative FACS profiles and quantification of Lin^−^ BM cells of six non-Tg mice stained for CD11c and gated on the same cell surface markers illustrated in panel A. CD11b^+^ spleen cells were included as controls. (K) Western blot analysis for Nef expression in myeloid cells of Tg mice. CD11b^+^ Gr1^+^ cells and DC were purified from non-Tg and CD11c/Nef Tg mice and their proteins extracted and processed for detection of Nef with anti-Nef antibody. Membrane was stripped and reprobed with anti-actin antibody. *, *P* ≤ 0.05; **, *P* ≤ 0.01; ***, *P* ≤ 0.001.

For our analysis, gating was performed on lin^−^ cells (<5%) (Fig. 4A) in BM (Fig. 4B), blood, and spleen (Fig. 4C) cells of non-Tg and CD11c/Nef Tg mice, along with a combination of other markers used to define specific hematopoietic precursors (Sca1, c-kit, CD34, and CD16/32). The percentages of LSK (lin^−^ Sca1^+^ c-kit^+^) (R7) and Sca1^−^ c-kit^+^ (myeloid/erythroid) progenitors were found to be higher in Tg BM (Fig. 4B). Committed myeloid GMP (Sca1^−^ c-kit^+^ CD34^+^ CD16/32^hi^) (R4) precursors were expanded and MEP (Sca1^−^ c-kit^+^ CD34^−^ CD16/32^−^) (R6) were decreased in all compartments of Tg mice (Fig. 4B and C), suggesting a skewed differentiation toward myeloid lineage at the expense of erythroid lineage in Tg mice. CMP (Sca1^−^ c-kit^+^ CD34^+^ CD16/32^low^) (R5) showed no difference in BM and spleen of Tg and non-Tg mice (Fig. 4B and C) but a modest increase in Tg PBMC (Fig. 4C).

When the same number (10^3^ cells) of cell-sorted purified Tg or non-Tg progenitor cells (GMP, CMP, or LSK) were plated in methylcellulose supplemented with interleukin-3 (IL-3), IL-6, SCF, and erythropoietin to induce their differentiation, comparable numbers of total CFU were generated (data not shown), suggesting their similar potential for differentiation on a per-cell basis. However, when total cells from BM, spleen, and blood were assayed, the number of CFU generated *in vitro* was significantly higher in Tg than in non-Tg compartments (Fig. 4D), indicating a higher number of Tg hematopoietic precursors. The majority of more numerous Tg colonies were more condensed and bigger than non-Tg ones (Fig. 4E) and represented CFU-GM, CFU-M, and CFU-G (Fig. 4F), indicating the presence of higher numbers of Tg myeloid precursors and suggesting their increased proliferation. To test this possibility, CFU-GM were picked, stained with CFSE, and replated in methylcellulose. FACS analysis of CFSE dilution (reflecting the number of cell divisions) demonstrated higher proliferation of Tg than non-Tg CFU-GM (Fig. 4G). In addition, BM cells cultured in the presence of IL-4 and GM-CSF, to differentiate them into DC, showed lower percentages of Tg DC (CD11c^+^ CD11b^+^) and higher Tg CD11b^+^ Gr1^+^ cell production relative to non-Tg controls (Fig. 4H), suggesting skewed differentiation of DC precursors. Erythroid colonies, generated in Tg cultures of total BM cells or of purified CMP, were also decreased relative to those in control non-Tg cultures (Fig. 4I), consistent with decreased MEP observed *ex vivo* by FACS analysis (Fig. 4B and C). Together, these results suggest that the higher number of Tg CD11b^+^ Gr1^+^ cells originates from a higher number of proliferating myeloid GMP.

To determine whether the expansion of myeloid progenitors in CD11c/Nef Tg mice could result from Nef expression in these cells, we measured cell surface CD11c expression on different myeloid cells by FACS, because the promoter of the CD11c gene is driving expression of *nef* in these Tg mice. We confirmed that early myeloid progenitors (GMP and CMP) express only low levels of CD11c on a small percentage of progenitors (Fig. 4J), as previously reported (22). However, Western blot analysis on the more abundant myeloid cell subsets showed that Nef is expressed in DC (CD11c^+^) but not in CD11b^+^ Gr1^+^ neutrophils (Fig. 4K), as expected, since the latter are CD11c negative.

Therefore, it appears that the accumulation of CD11b^+^ Gr1^+^ cells in CD11c/Nef Tg mice is not a consequence of Nef expression in their precursors but is indirect and not cell autonomous.

### The CD11b^+^ Gr1^+^ cell accumulation in CD11c/Nef Tg mice is stroma independent.

**FIG 5:**
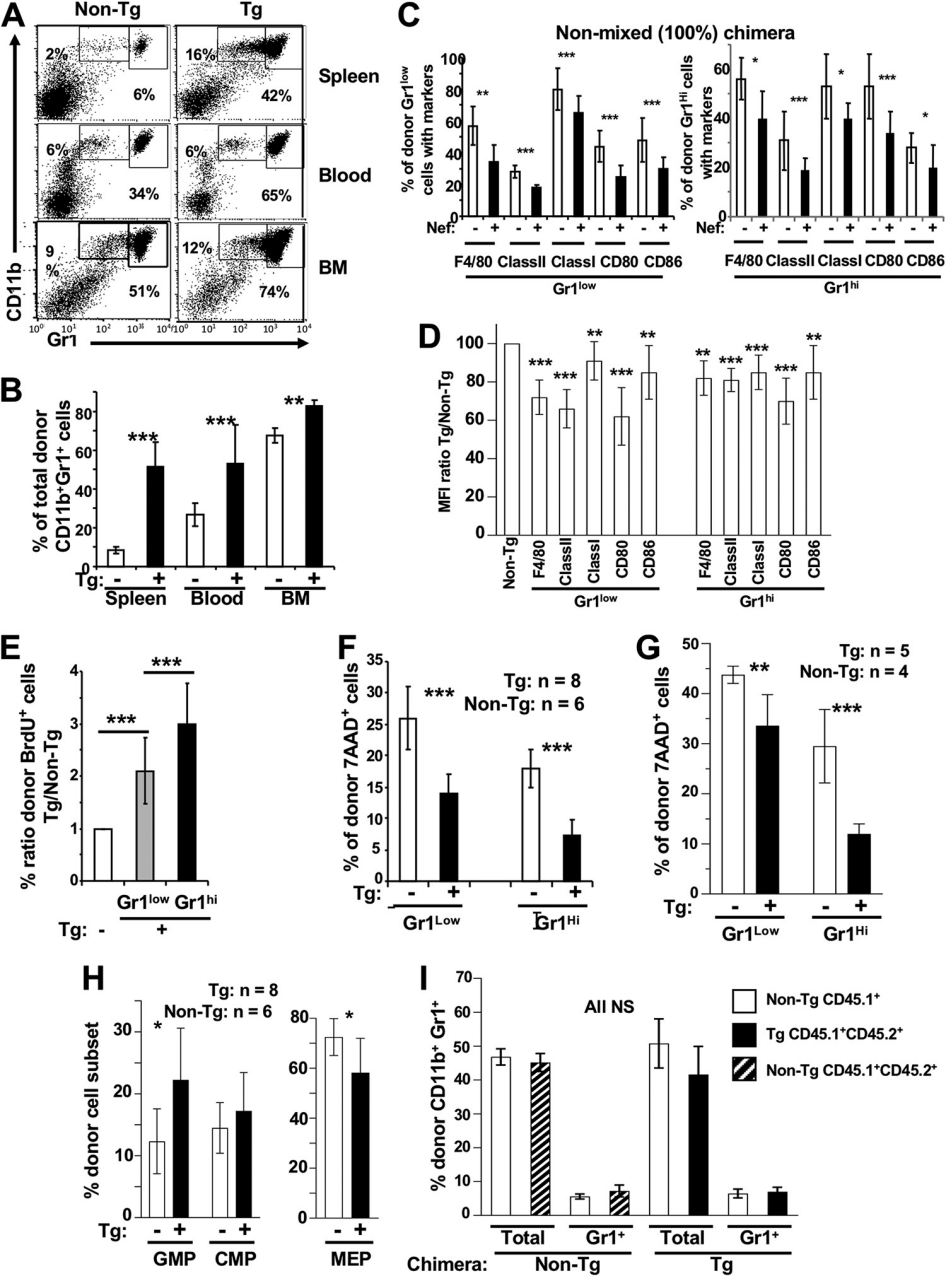
Enhanced CD11b^+^ Gr1^+^ cell phenotype of CD11c/Nef Tg mice can be observed in recipient mice transplanted with Tg fetal liver (FL) cells. Lethally irradiated non-Tg CD45.2^+^ mice were transplanted with FL cells from CD45.1^+^ CD45.2^+^ Tg or non-Tg E14.5 embryos (A to H) or with mixed (1:1) CD45.2^+^/CD45.1^+^ Tg and non-Tg or control CD45.1^+^ non-Tg FL cells (I). Recipient mice were analyzed 3 to 5 months after transplantation. (A and B) Representative FACS profiles (A) and percentage of donor CD45.1^+^ CD11b^+^ Gr1^+^ cells (B) in spleen, blood, and BM of recipient mice (*n* = 7 Tg and 5 non-Tg). (C and D) Nonmixed (100%) FL cell transplanted mice. (C) Percentages are of donor spleen CD45.1^+^ CD45.2^+^ CD11b^+^ Gr1^+^ (Gr1^low^ and Gr1^hi^) cells expressing F4/80, MHC class I, MHC class II, CD80, and CD86 in recipient mice (*n* = 6 to 12) after FL cell transplantation. (D) Mean fluorescence intensity (MFI) of the indicated markers in donor CD45.1^+^ CD11b^+^ Gr1^low^ and in CD11b^+^ Gr1^hi^ cell subsets of recipient mice is shown. (E) Proliferation of sorted-purified donor CD11b^+^ Gr1^+^ spleen cells from non-Tg (*n* = 4) and Tg (*n* = 5) mice assessed by BrdU labeling. (F and G) Survival of donor CD45.1^+^ CD11b^+^ Gr1^+^ spleen cells *ex vivo* (F) and after their purification and cultured *in vitro* in the presence or not of LPS (1 μg/ml) for 24 h (G) was assessed by FACS with 7-AAD labeling. (H and I) Donor (CD45.1^+^ CD45.2^+^) BM progenitors from nonmixed chimera (*n* = 6 non-Tg and 8 Tg) (H) or donor (CD45.1^+^ and CD45-1^+^ CD45-2^+^) CD11b^+^ Gr1^+^ cells in spleen of mixed chimeric mice (*n* = 6 non-Tg and 6 Tg chimera) (I) were analyzed by FACS and percentages calculated as for Fig. 4A. NS, not significant. Results are from 3 independent experiments for all phenotypes. *, *P* ≤ 0.05; **, *P* ≤ 0.01; ***, *P* ≤ 0.001.

We used a transplantation strategy to study the role of the stroma in Tg myeloid cell accumulation. Donor fetal liver (FL) cells (containing large numbers of hematopoietic stem cells) were from Pep3b (CD45.1^+^) C3H (CD45.2^+^) Tg or non-Tg mice. These mice harbor the Pep3b marker to distinguish donor from host cells. FL cells were transplanted into lethally irradiated normal (CD45.2^+^) C3H hosts. In both Tg and non-Tg reconstituted chimeras studied 3 to 5 months later, ∼90 to 95% of the hematopoietic cells were of donor origin, i.e., CD45.1^+^ CD45.2^+^. In chimeric mice reconstituted with Tg cells, an accumulation of donor-derived CD45.1^+^ CD45.2^+^ CD11b^+^ Gr1^+^ cells was observed in BM, spleen, and blood (Fig. 5A and B). Among these, a lower percentage and mean fluorescent intensity (MFI) of maturity markers were observed (Fig. 5C and D). CD4^+^ and CD8^+^ T cells also were decreased (data not shown). Proliferation of spleen CD11b^+^ Gr1^+^ cells of chimeric mice, measured by BrdU incorporation *in vivo*, was enhanced (Fig. 5E). Spontaneous (Fig. 5F) or lipopolysaccharide (LPS)-induced (Fig. 5G) apoptosis/death of Tg relative to non-Tg CD11b^+^ Gr1^+^ donor cells was decreased. FACS analysis showed that the number of donor myeloid Tg GMP in BM was increased, whereas MEP were decreased (Fig. 5H).

These phenotypes are very similar to those described above for CD11b^+^ Gr1^+^ cells in CD11c/Nef Tg mice themselves, indicating that the accumulation of CD11b^+^ Gr1^+^ cells in CD11c/Nef Tg mice can be independent of the stroma.

### The accumulation of CD11b^+^ Gr1^+^ cells in CD11c/Nef Tg mice is abrogated in the presence of non-Tg hematopoietic cells.

The apparent skewed differentiation of purified myeloid Tg progenitors *in vitro* (Fig. 4H and I) suggests that they are impaired. To test this hypothesis, we compared their reconstitution potential *in vivo* with that of non-Tg progenitors in the same mouse. For this, we used a transplantation strategy similar to that described above, except that mixed (1:1) FL cells from Tg (CD45.1^+^ CD45.2^+^) and non-Tg (CD45.1^+^) mice were used as donors. The control represented mixed (1:1) FL cells from CD45.1^+^ non-Tg and CD45.1^+^ CD45.2^+^ non-Tg mice. Analysis was performed as described above 4 to 5 months posttransplantation by gating on donor CD45.1^+^ or CD45.1^+^ CD45.2^+^ cells. No accumulation of donor Tg CD11b^+^ Gr1^+^ cells (relative to non-Tg ones) was observed in any of the organs tested (Fig. 5I and data not shown), indicating that the CD11b^+^ Gr1^+^ cell accumulation in Tg mice can be abrogated by the presence of non-Tg hematopoietic cells. This result suggests that donor Tg myeloid precursors were outperformed by non-Tg ones in a context of transplantation.

### The accumulation of CD11b^+^ Gr1^+^ cells is also present in CD4C/Nef Tg mice expressing Nef through the regulatory elements of the human CD4 gene.

**FIG 6:**
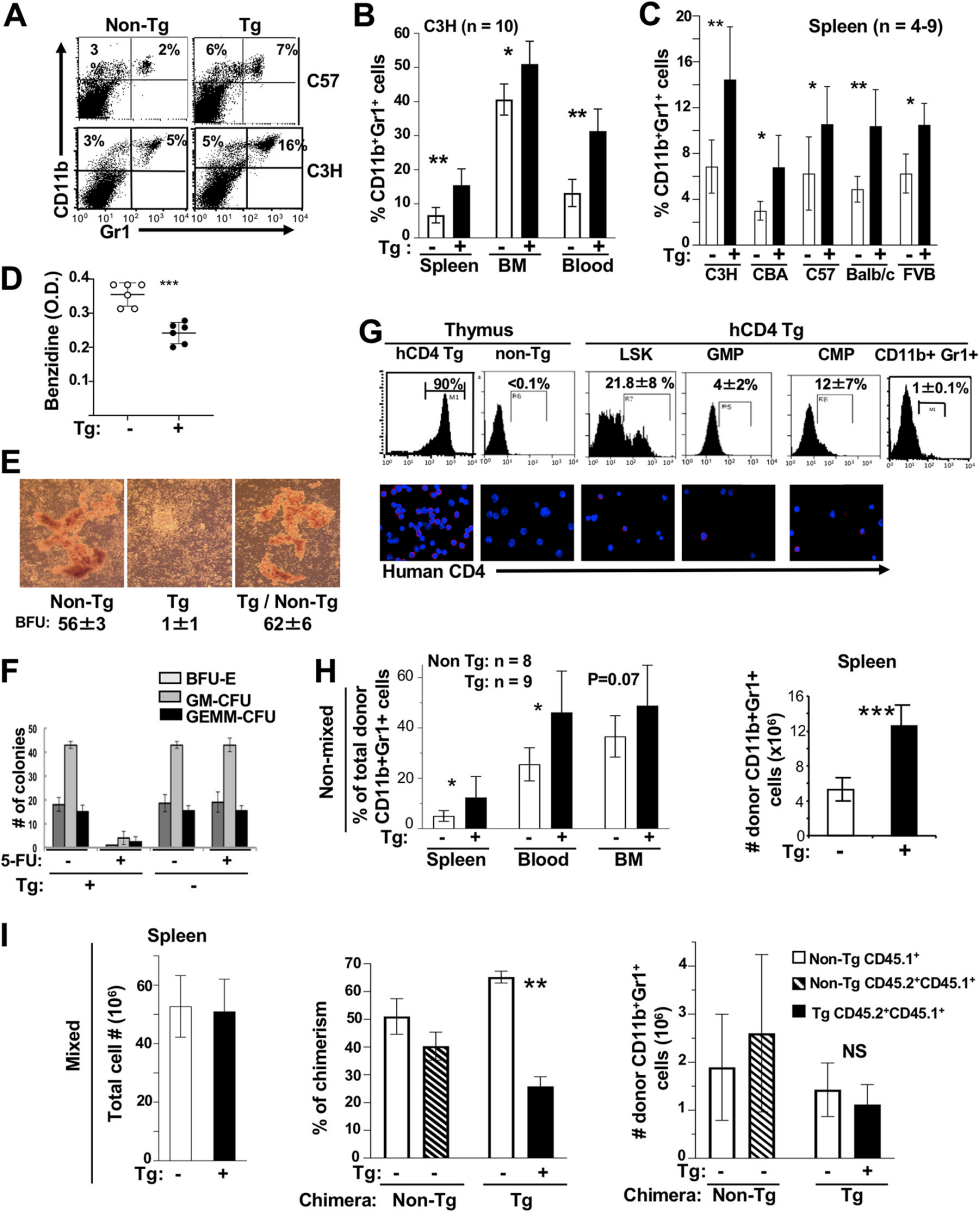
Accumulation of CD11b^+^ Gr1^+^ cells in CD4C/Nef Tg mice. (A to C) Representative FACS profiles (A) and quantification (B and C) of CD11b^+^ Gr1^+^ cells in indicated organs of Tg and non-Tg mice of different backgrounds. Tg mice were bred for at least six generations as heterozygous on each mouse background before being studied. C57, C57BL/6. (D) Evaluation of benzidine-positive cells in BM of Tg and non-Tg mice. Total BM cells (1 × 10^6^) were suspended and stained with benzidine for 5 min and lysed, and the optical density at 420 nm (OD_420_) was measured. ***, *P* ≤ 0.001. (E) BM cells from control and CD4C/HIV^MutA^ Tg mice or mixed mice (Tg/non-Tg) (1:1) were incubated with hydrocortisone (10^−6^ M) in methylcellulose and BFU-E colonies counted. (F) Impaired BM recovery after 5-FU treatment. Control non-Tg and CD4C/HIV^MutA^ Tg mice were treated with 5-FU (100 mg/kg body weight) and sacrificed 10 days later. BM cells were cultured in methylcellulose and the number of colonies counted. (G) Expression of reporter human cell surface CD4 (hCD4) in myeloid progenitors and Gr1^+^ splenocytes of CD4/hCD4 Tg mice (*n* = 4). Expression in Tg and non-Tg thymus served as a positive and negative control, respectively. Myeloid progenitors and controls were labeled as shown in Fig. 4A and analyzed by FACS (upper) or by microscopic immunofluorescence (lower). (H and I) Non-Tg CD45.2 mice were lethally irradiated and transplanted, as described for Fig. 5I, with FL cells from CD45.1^+^ CD45.2^+^ Tg or non-Tg E14.5 embryos (nonmixed) (H) or with mixed (1:1) CD45.1^+^ CD45.2^+^ Tg/CD45.1^+^ non-Tg or control CD45.1^+^ CD45.2^+^ non-Tg/CD45.1^+^ non-Tg FL (I) cells. Recipient mice were analyzed 6 months posttransplantation. Results are from 3 independent experiments. *, *P* ≤ 0.05; **, *P* ≤ 0.01; ***, *P* ≤ 0.001.

To determine whether similar accumulation of CD11b^+^ Gr1^+^ cells would be observed in Tg mice expressing Nef in the same cells as those expressing CD4 in humans (myeloid cells, such as monocytes, macrophages, DC [23], and some of their progenitors [24–26]), we studied the CD4C/Nef Tg mice that express Nef under the regulatory elements of the human CD4 gene (14). We already reported that myeloid CD11c^+^ CD11b^hi^ DC accumulate in spleen of these older CD4C/Nef Tg mice (16), as they also do in CD11c/Nef Tg mice (17). Analysis of C3H CD4C/Nef Tg mice showed that the percentage of CD11b^+^ Gr1^+^ cells was higher in blood, spleen, and BM than in their non-Tg littermates (Fig. 6A and B). This was observed in Tg mice bred on five different backgrounds (C3H/HeNHsd, C57BL/6, CBA, BALB/c, and FVB) (Fig. 6C) and was most evident in older Tg mice (data not shown), as in CD11c/Nef Tg mice discussed above (Fig. 1I). However, myeloid cell accumulation appeared to be less extensive in CD4C/Nef than in CD11c/Nef Tg mice.

Myeloid cell accumulation has not been studied in CD4C/HIV^Wt^ Tg mice expressing all HIV genes. We reported that the latter Tg mice develop numerous multisystem phenotypes undistinguishable from those of CD4C/Nef Tg mice (14, 27). It remains unknown whether another HIV gene(s) collaborates with Nef for promoting myeloid cell accumulation.

Interestingly, however, such myeloid cell accumulation was not observed in CD4C/HIV^MutH^ Tg mice (data not shown) expressing all HIV genes except *nef* (14), indicating that Nef is necessary and sufficient to elicit this phenotype.

We next investigated the status of BM cells in CD4C/Nef Tg mice. We found that the number of more mature benzidine-positive erythroid BM cells was reduced in CD4C/Nef Tg mice (Fig. 6D), suggesting impaired erythroid cell differentiation. In addition, colony assay in methylcellulose was performed with these BM cells. Results showed an enhanced number of total BM colonies relative to those from non-Tg mice (122.5 ± 16.3 versus 89.5 ± 7.8). However, Tg colonies first grown in methylcellulose, followed by incubation in suspension with GM-CSF and then replated in methylcellulose with GM-CSF to further differentiate them, were not capable of full differentiation in the presence of GM-CSF and remained undifferentiated as CFU-GM (non-Tg, <1; Tg, 53 ± 2 colonies). Tg early erythroid BFU-E colonies were also severely depleted (Fig. 6E), apparently in a cell-autonomous way (Table 1), likely explaining the lower number of more mature benzidine-positive Tg BM erythroid cells observed (Fig. 6D). Finally, we evaluated the capacity of the BM from these Tg mice to recover from a severe insult, a method frequently used to assess the integrity of hematopoietic progenitors. We challenged these Tg mice with the drug fluorouracil (5-FU), toxic for BM, and analyzed their BM progenitors 10 days later. Normal non-Tg mice recovered relatively well from this treatment, showing a number of colonies similar to that from untreated non-Tg mice (Fig. 6F). However, a severe failure to recover was evident in BM of 5-FU-treated Tg mice, both morphologically (data not shown) and by the low number of BFU-E, GM-CFU, and GEMM-CFU colonies generated relative to those from 5-FU-treated non-Tg mice (Fig. 6F), strongly suggesting impairment of myeloid and erythroid progenitor differentiation.

**TABLE 1:** Impaired *in vitro* differentiation of erythroid precursors from CD4C/HIV Tg mice^*a*^

BM progenitor	Stroma	No. of BFU-E
Non-Tg	Non-Tg	49 ± 7
	Tg	54 ± 6
	Non-Tg/Tg	51 ± 5
Tg	Non-Tg	0
	Tg	0
	Non-Tg/Tg	0

a BM cells from control and CD4C/HIV^MutA^ Tg mice were incubated with hydrocortisone (10^−6^ M) in the presence of stromal cells from non-Tg or Tg mice either alone or mixed (non-Tg/Tg) (1:1) and BFU-E colonies counted.

We next investigated whether the expansion of myeloid progenitors and the severe BM failure observed in CD4C/Nef Tg mice result from Nef expression in these progenitor cells through the CD4C regulatory elements. We already reported that HIV or reporter human CD4 is expressed in mature myeloid cells (macrophages and DC) of CD4C/HIV or CD4C/hCD4 Tg mice (28). For this experiment, we used CD4C/hCD4 and CD4C/hCD4^+^ CCR5 Tg mice and measured the cell surface expression of the reporter human CD4 protein (hCD4) by FACS and immunofluorescence analyses. In these two founder lines, the reporter was expressed in LSK and in early myeloid progenitors (LSK>CMP>GMP) (Fig. 6G and data not shown), but hCD4 was minimally expressed in CD11b^+^ Gr1^+^ cells (Fig. 6G), as expected. These results show that the regulatory elements of the human CD4 gene are active in mouse myeloid and early (LSK) cell progenitors, consistent with expression of CD4 in human myeloid progenitors (24, 26).

Therefore, these results suggest that Nef expression in early and myeloid progenitors of CD4C/Nef Tg mice has direct (cell-autonomous) effects. It is likely responsible for the severe failure of BM recovery observed in these Tg mice following 5-FU challenge. Such Nef expression in BM myeloid precursors, alone or with Nef-expressing DC (16), may favor the accumulation of CD11b^+^ Gr1^+^ cells, at the expense of erythroid differentiation, in these CD4C/Nef Tg mice.

### The accumulation of CD11b^+^ Gr1^+^ cells in CD4C/Nef is transplantable but is abrogated in the presence of non-Tg hematopoietic cells.

To determine whether the accumulation of CD11b^+^ Gr1^+^ cells in CD4C/Nef Tg mice was stroma dependent, we performed FL cell transplantation. FL cells from CD45.1^+^ CD45.2^+^ CD4C/Nef Tg mice or from control non-Tg littermates were transplanted into lethally irradiated normal C3H hosts and sacrificed 3 to 4 months posttransplantation. An accumulation of donor-derived CD11b^+^ Gr1^+^ cells was observed in spleen and blood (but not in BM) of Tg relative to non-Tg chimera (Fig. 6H), indicating that this phenotype is stroma independent, as found in CD11c/Nef Tg mice (Fig. 5A and B).

We also performed a similar transplantation experiment with FL cells from non-Tg or Tg mice (CD45.1^+^ CD45.2^+^) mixed (1:1) with FL cells of non-Tg (CD45.1^+^) mice as donors. No accumulation of donor CD11b^+^ Gr1^+^ cells was observed relative to non-Tg controls in spleen (Fig. 6I), BM, or blood (data not shown). This result indicates that the accumulation of CD11b^+^ Gr1^+^ Tg cells can be abrogated by non-Tg hematopoietic cells, as in CD11c/Nef Tg mice (Fig. 5I), consistent with the 5-FU data (Fig. 6F) showing that their progenitors are impaired.

### Nef-induced CD11b^+^ Gr1^+^ myeloid cell accumulation is associated with enhanced levels of G-CSF mediated by IL-17.

**FIG 7:**
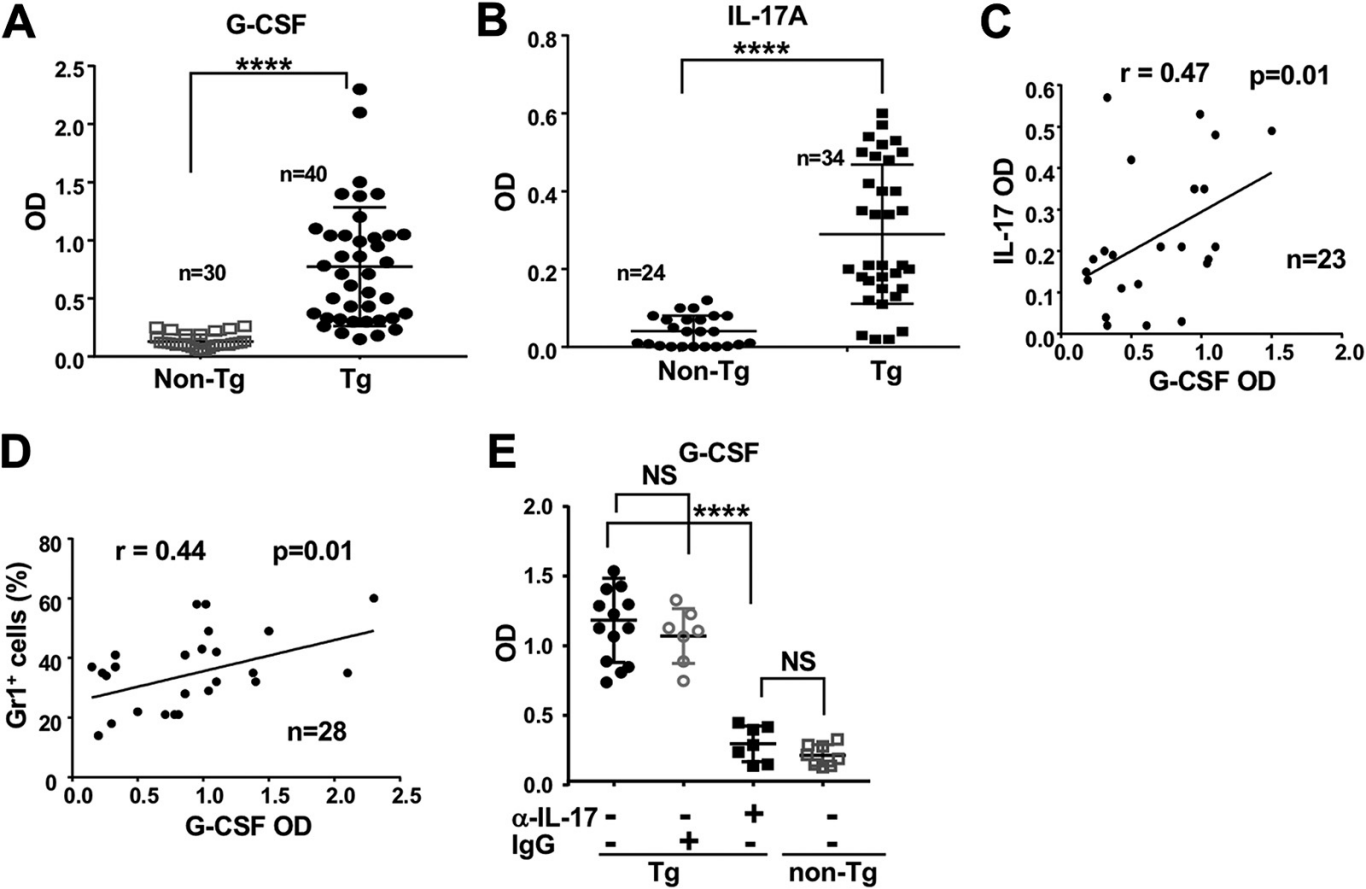
Enhanced IL-17 and G-CSF levels in sera of CD11c/Nef Tg mice. (A and B) G-CSF (A) and IL-17 (B) were measured in sera of Tg and non-Tg mice by ELISA. (C and D) Correlation between levels of both cytokines (C) and between the percentage of Gr1^+^ cells expanded and levels of G-CSF (D) was evaluated with the Spearman’s test. (E) G-CSF levels in serum of Tg and non-Tg mice 7 days after receiving anti-IL-17RA (α-IL-17) antibody or control irrelevant IgG (IgG) i.p. Results from 4 independent experiments with a total of 7 mice in each group treated with IgG or anti-IL-17RA. ****, *P* ≤ 0.0001.

The very low CD11c (Fig. 4J) and undetectable Nef (Fig. 4K) expression in BM progenitors of non-Tg mice and in CD11b^+^ Gr1^+^ cells of CD11c/Nef Tg mice suggested that the latter cells accumulate through a non-cell-autonomous mechanism, such as cytokine production by other cells. Among these, granulocyte colony-stimulating factor (G-CSF) is a well-known mediator of myeloid cell expansion (29, 30), in some cases through IL-17 (31). We measured these two cytokines in serum of non-Tg and CD11c/Nef Tg mice and found both of them to be significantly increased in Tg mice (Fig. 7A and B) and correlated (Fig. 7C). The levels of G-CSF also correlated with the proportion of CD11b^+^ Gr1^+^ cells in the blood (Fig. 7D).

To determine whether higher IL-17 levels cause the enhanced G-CSF production, we neutralized IL-17 *in vivo* by injecting anti-IL-17A antibody (Ab) into a group of CD11c/Nef Tg mice and measured G-CSF levels 7 days after the initiation of this treatment. The levels of G-CSF were significantly reduced in anti-IL-17RA-treated Tg mice but not in Tg mice treated with irrelevant IgG (Fig. 7E), indicating a positive control of IL-17 on G-CSF production and suggesting that G-CSF contributes to the expansion of CD11b^+^ Gr1^+^ myeloid cells in these mice.

### Src deletion favors, but Hck/Lyn deficiency abrogates, the Nef-mediated expansion of CD11b^+^ Gr1^+^ myeloid cells of CD4C/Nef Tg mice.

**FIG 8:**
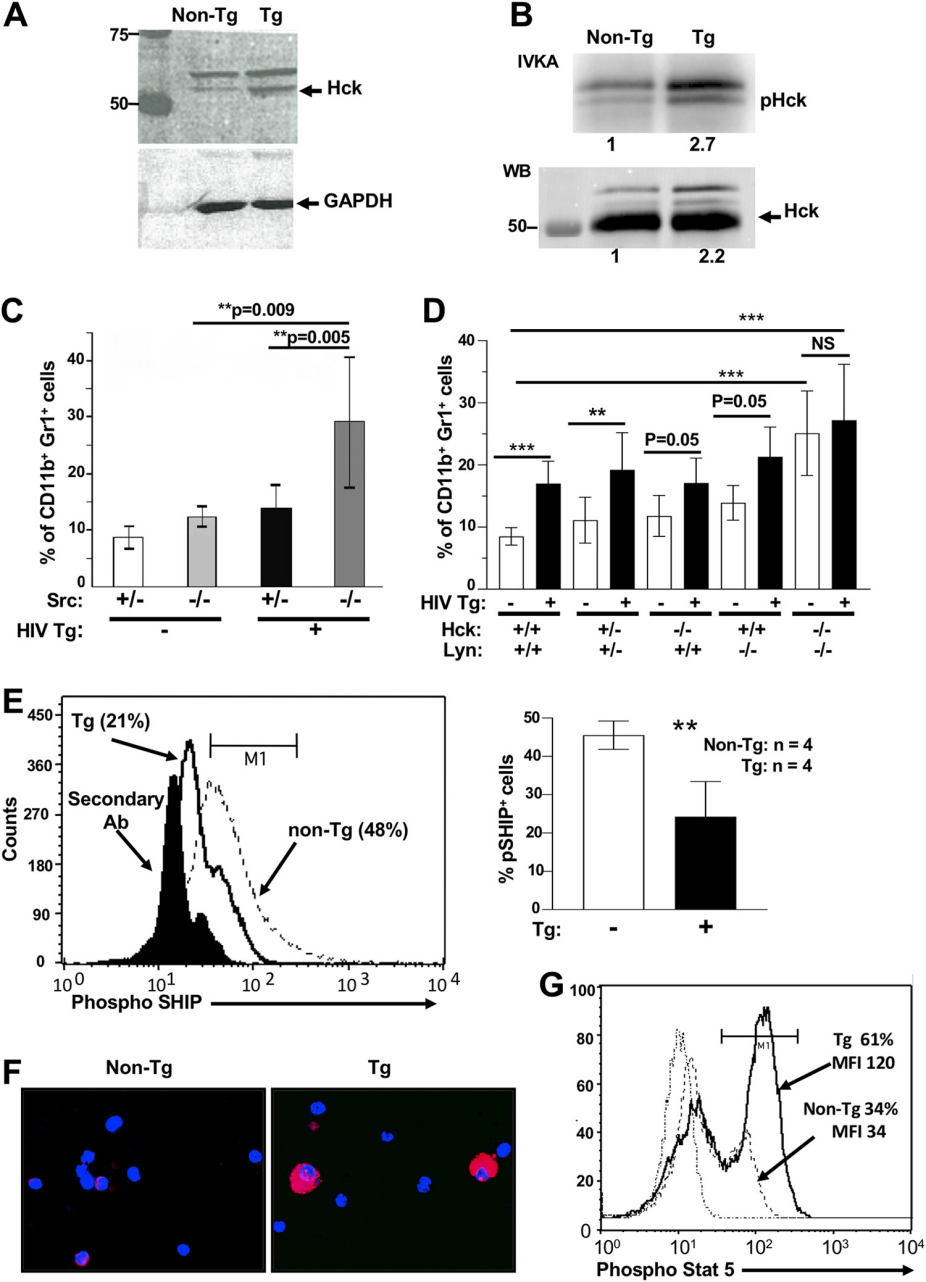
Role of Src-related kinases in accumulation of CD11b^+^ Gr1^+^ myeloid cells of HIV Tg mice. (A and B) Lysates of spleen cells from CD11c/Nef Tg mice were analyzed by Western blotting (WB) with anti-Hck (A) or immunoprecipitated with anti-Hck Ab, and these immunoprecipitates were subjected to IVKA (B). Numbers below the graph refer to relative intensity. (C and D) Evaluation of percentage of CD11b^+^ Gr1^+^ cell subset in spleen of CD4C/Nef and CD4C/HIV^MutA^ Tg mice bred on a wild-type (+/+), heterozygote (+/−), or homozygote (−/−) Src (*n* = 4 to 8/group) (C) or Hck/Lyn (*n* = 3 to 12/group) (D) gene-deficient background. (E) Evaluation of p-SHIP^Y1060^ levels in unplated, freshly isolated purified peritoneal macrophages of Tg and non-Tg mice. Cells were labeled intracellularly with anti-p-SHIP^Y1060^ Ab and Alexa F633-labeled secondary Ab, analyzed by FACS (left), and results quantitated (right). Results are from four experiments. (F and G) Evaluation of pStat-5^Y694^ levels in CD11b^+^ Gr1^+^ cells of Tg and non-Tg mice. (F) Spleen CD11b^+^ Gr1^+^ cells were purified, cytospotted, and subjected to immunofluorescence with anti-p-Stat-5^Y694^ Ab and Alexa F594-labeled secondary Ab. (G) Spleen cells were also labeled with anti-CD11c, CD11b, Gr-1, and the two Ab mentioned above and analyzed by FACS. Results are from 2 experiments with 2 mice per group in each experiment. **, *P* ≤ 0.01; ***, *P* ≤ 0.001.

Because Nef has been reported to bind to members of the Src family, in particular to Hck (32), and to activate them (33), we studied the involvement of these signaling molecules in the expansion of CD11b^+^ Gr1^+^ cells. First, Hck levels were measured by Western blot analysis (Fig. 8A), and Hck activity was evaluated by an *in vitro* kinase assay (IVKA) (Fig. 8B). Both were found to be slightly elevated in Tg cells, confirming the activating role of Nef for Src kinases reported in other cells (33, 34).

Next, we bred Tg mice on Src family gene-deficient backgrounds. Intriguingly, in CD4C/Nef Tg mice harboring homozygous (−/−) deletion of Src, the expansion of CD11b^+^ Gr1^+^ cells was larger than that in control heterozygous (+/−) Src Nef Tg mice or than in non-Tg mice (Fig. 8C), suggesting that Src signaling inhibits Nef activity. This could occur either directly in myeloid cells (possibly through its binding to Src) or indirectly in other cells not expressing Nef but involved in the accumulation of CD11b^+^ Gr1^+^ cells. Finally, we bred HIV Tg mice on an Hck/Lyn double gene-deficient background. It is already known that mice deficient for Hck and Lyn show an accumulation of immature myeloid cells (7) very similar to that of the CD11c/Nef and CD4C/Nef Tg mice shown above. We found that deletion of Hck/Lyn induced expansion of CD11b^+^ Gr1^+^ cells in non-Tg double Hck/Lyn knockout (KO) mice (Fig. 8D), confirming an earlier report (7). However, the further HIV-mediated expansion of these cells was prevented in homozygous (−/−) double Hck/Lyn gene-deficient HIV Tg mice, but not in heterozygous (+/−) or single gene-deficient HIV Tg mice (Fig. 8D), strongly suggesting that Nef favors myeloid cell expansion through Hck/Lyn.

Because CD4C/Nef Tg mice and Hck/Lyn double KO mice show similar accumulation of CD11b^+^ Gr1^+^ cells and because the loss of Hck/Lyn was reported to be associated with decreased phospho-SHIP (pSHIP) and enhanced p-Stat-5 (7), we measured these effectors. We found that myeloid cells from Nef Tg mice show decreased pSHIP (Fig. 8E) and increased p-Stat-5 (Fig. 8F and G), as reported in Hck/Lyn KO mice (7), suggesting that loss of Hck/Lyn and Nef function share a common molecular pathway.

## DISCUSSION

Our data show that HIV-1 Nef, expressed in Tg mice, can trigger the progressive stroma-independent accumulation of CD11b^+^ Gr1^+^ granulocytic cells and MDSC, most likely through their increased proliferation and resistance to death as well as expansion of their progenitors. The MDSC of Nef Tg mice exhibit the same characteristics as those generated in other diseases (5): they are immature and show iNOS-mediated immunosuppression against T cells. They may have contributed to the impaired control of oral C. albicans infection previously reported in CD11c/Nef Tg mice (17).

### Apparent role of DC in Nef-induced CD11b^+^ Gr1^+^ cell expansion.

Two Tg mouse models were investigated here, CD11c/Nef and CD4C/Nef Tg mice. In CD11c/Nef Tg mice, we documented that Nef is mainly expressed in mature DC (17) (Fig. 4K), and this promoter is known to also be active in precursor pre-cDC and, to a lesser extent, in pDC (22). CD4C/Nef Tg mice express Nef in several cell subsets, such as CD4^+^ thymocytes, mature CD4^+^ T cells, and macrophages, but also in DC and pDC (16). Since DC from both Nef Tg strains express *nef* (16, 17), Nef-expressing Tg DC or pDC are likely to be involved in the accumulation of CD11b^+^ Gr1^+^ cells in these mice. Indeed, a similar accumulation of CD11b^+^ Gr1^+^ cells was observed after depletion of CD11c-positive DC or pDC in CD11c/DtA Tg mice (35–37). This cell accumulation was stroma-independent, abrogated in mixed chimera (35, 36) (as in Nef Tg mice) and thought to result from depletion of DC (35) or pDC (37). Inhibition of DC differentiation by S100A9 also was found to induce accumulation of MDSC (38). A similar phenomenon as in Nef Tg mice was also observed in SIV-infected macaques. After experimental deletion of CD4^+^ T cells, it was reported that a very large number of macrophages and of HLA-DM^+^ antigen-presenting cells (APC) are infected (39, 40) and that classical (CD14^+^ CD16^−^) and proinflammatory (CD14^+^ CD16^+^) monocytes accumulate (39). Interestingly, among these infected APC, some are likely to be DC known to harbor the HLA-DM marker (41, 42), suggesting that infected DC contribute to myeloid cell expansion in this SIV model, as seems to be the case in Nef Tg mice.

### Involvement of IL-17/G-CSF in Nef-induced CD11b^+^ Gr1^+^ cell expansion.

The accumulation of CD11b^+^ Gr1^+^ cells in CD11c/DtA Tg mice depleted of their DC or pDC was reported to be through enhanced expression of Flt3 ligand (35), MCP-1 (37), or elevated IL-17A-producing cells (36). Similarly, in Nef Tg mice, the presence of immature, poorly differentiated and/or functionally impaired (16, 17) Nef-expressing DC or pDC may cause non-cell-autonomous (bystander) accumulation of CD11b^+^ Gr1^+^ cells. Consistent with this notion, we documented enhanced IL-17 levels in serum of CD11c/Nef Tg mice, as reported by some groups for SIV (43) and HIV (44) infection. Activated DC have been shown to stimulate the production of IL-17 by other cells (45, 46). IL-17 is known to favor the induction of MDSC and of cells of the granulocytic lineage, both in mouse and human (47–50), through G-CSF (31). Here, we showed that the high levels of IL-17 were responsible for the increased levels of G-CSF in Nef Tg mice, indicating that the IL-17/G-CSF axis (31) is maintained despite the presence of Nef. G-CSF is elevated in HIV-infected subjects (51, 52) and is well known for its activity promoting granulopoiesis, erythroid suppression, and MDSC expansion (29, 30, 53), all observed in Nef Tg mice. Thus, IL-17/G-CSF are likely to be involved in the bystander accumulation of both subsets of CD11b^+^ Gr1^+^ cells in these Tg mice. However, we cannot ruled out that other pathways, not investigated here, such as a bystander effect of Nef secreted in exosomes, contribute to this phenotype.

### Other cell subset(s) may be involved in Nef-induced CD11b^+^ Gr1^+^ cell expansion in Tg mice.

Additional cell subset(s) may contribute to myeloid cell accumulation in CD4C/Nef Tg mice, since Nef is expressed not only in DC or pDC but also in T cells and other cells of the myeloid lineage, such as macrophages, Kupffer cells (14, 17), and osteoclasts (54), which are also targets of HIV or SIV infection. Indeed, we found here, using the human CD4 reporter Tg mice, that this CD4C promoter is also quite active in a fair percentage of early hematopoietic progenitors and more weakly active in a small proportion of myeloid progenitors (Fig. 6G), consistent with the reported low expression of CD4 in a small fraction of human BM progenitors (24, 26). However, in human patients, it has remained controversial (reviewed in references 13, 55, and 56) whether HIV can infect hematopoietic progenitors (26, 57) or not (58, 59). Independent of this controversy, the aberrant growth/differentiation of CD4C/Nef Tg myeloid colonies in clonogenic assays and the documented activity of the CD4C promoter in some early and myeloid progenitors suggest that part of the MDSC accumulation in CD4C/Nef Tg mice results from expression of Nef in myeloid precursors through a cell-autonomous process.

Interestingly, in both Nef Tg strains, the accumulation of CD11b^+^ Gr1^+^ cells was abrogated in mixed Tg–non-Tg chimera, a phenomenon also reported in mixed CD11c/DtA/WT chimera (35, 36). This result indicates that the biology of transplantable Nef-expressing Tg myeloid cells (possibly DC or pDC and their precursors) can be modified by the presence of transplantable non-Tg hematopoietic cells. The nature of this Tg/non-Tg cell interaction remains unclear. Tg early or myeloid precursors may be defective in critical myeloid cell differentiation signal(s) and may be outcompeted by non-Tg cells. This scenario is most likely occurring in CD4C/Nef Tg mice in which BM regeneration is severely impaired (Fig. 6E and F). Alternatively, non-Tg cells may inhibit Tg cell survival or function. The mechanisms behind this intriguing result may help to explain, at least partly, some features of human AIDS, notably the impressive success of BM transplantation in some HIV^+^ patients and the late appearance of myeloid cell expansion and of other myeloid-related phenotypes, such as neuroAIDS. Indeed, CD163^+^ perivascular macrophages infiltrating the CNS were reported to be derived from circulating CD16^+^ monocytes (13). Early in AIDS and especially after BM transplantation, the high ratio of normal versus HIV-infected hematopoietic cells may prevent myeloid cell expansion, either directly as in non-Tg/Tg chimera or by preventing their infection, as shown in CD4^+^ T cell-depleted SIV-infected macaques (39, 40). When this ratio decreases, higher number of myeloid cells, especially DC (as documented for APC in CD4^+^ T cell-depleted SIV-infected macaques [40]) and/or hematopoietic progenitors may get infected, and myeloid cell expansion may proceed.

### Nef-induced CD11b^+^ Gr1^+^ cell expansion mediated through Hck/Lyn.

Nef binds to and activates members of the Src kinase family, preferentially Hck (33). We previously reported that Lck (another Src kinase) is activated by Nef in thymocytes of CD4C/Nef Tg mice and that these cells were depleted (34). Similarly, in CD11c/Nef Tg mice, Hck activity was found to be increased in spleen cells (Fig. 8B), consistent with the activating effect of Nef on Hck in other cells (33) and on Lck in human T cells and in T cells of CD4C/Nef Tg mice (34). Moreover, enhanced Stat-5 phosphorylation was documented in Nef Tg mice (Fig. 8F and G), possibly as a result of increased Hck activity, capable of phosphorylating Stat-5 (60). In addition, deletion of Hck/Lyn abrogates Nef-mediated myeloid cell expansion in these double Hck/Lyn knockout Nef Tg mice (Fig. 8D), indicating that these effectors are required for Nef functions. Thus, it is likely that the observed constitutive expression of activated Hck (and likely that of Lyn) in Nef Tg mice is contributing to myeloid cell expansion in these Tg mice. The generation of Tg mice expressing activated Hck/Lyn through the same regulatory elements (CD11c and CD4C) as those used in Nef Tg mice would be helpful to validate this apparent gain-of-function mechanism.

It is also possible that Hck/Lyn behaves as a loss of function regarding Tg CD11b^+^ Gr1^+^ accumulation despite being activated by Nef. Indeed, Nef Tg mice show phenotypes very similar to those of double Hck/Lyn-deficient mice, i.e., a stroma-independent accumulation of CD11b^+^ Gr1^+^ cells and myeloid progenitors, decreased pSHIP, and increased p-Stat-5 levels in myeloid cells, as reported before (7) and confirmed here (Fig. 8D to G).

Genetic Hck rescue experiments, as we did with Lck in T cells (34), may clarify which of these two possibilities is more likely to explain the phenotypes observed in Nef Tg mice.

### Are these mouse phenotypes relevant to human disease?

As models for human AIDS, these Nef Tg mice have obvious limitations. In contrast to HIV in humans, there is no virus replication or immune response against HIV. The *nef* gene also is expressed in a much larger number of DC (for CD11c/Nef and CD4C/Nef Tg) or of CD4**^+^** T cells (for CD4C/Nef Tg). The latter characteristic does not necessarily invalidate their relevance. Indeed, the very high number of myeloid cells expressing HIV genes is not unique to these Nef Tg models and also occurs during SIV infection, especially in macrophages (39, 61, 62). In one SIV model, based on anti-CD4 antibody-mediated CD4^+^ T cell depletion, ∼80% of productively infected cells are macrophages (39). In addition, in the latter model, a large number of microglial cells (39) and of HLA-MD^+^ APC become infected (40). Some of these APC are likely dendritic cells known to be positive for HLA-DM (41, 42).

Interestingly, despite these limitations, important features or phenotypes of HIV or SIV infection are replicated in our Tg mouse models. First, CD14^+^ CD16^+^ or other subsets of monocytes, sharing many of the characteristics of mouse CD11b^+^ Gr1^+^ cells (3), are expanded during HIV or SIV infection (8–12), including in the CD4^+^ T cell-depleted SIV model discussed above, expressing SIV in a very large percentage of myeloid cells (39), some of them likely DC (40), as in Nef Tg mice. Second, DC and pDC are infectable *in vitro* (63) and infected to some extent during HIV or SIV infection (64, 65), including in humanized mice (66) and very likely in the CD4-depleted SIV model where they were identified as HLA-DM**^+^** (40). Our results with CD11c/Nef Tg mice suggest that infection of DC favors the accumulation of myeloid cells, as we documented in these Tg mice. Third, anemia is one of the best correlates of disease progression in HIV infection (67). MDSC accumulation during HIV or SIV infection is associated with impaired erythroid cell differentiation (68), as we found in both Nef Tg strains. Fourth, increased vacuolization of circulating monocytes and BM granulocytic cells, which is a striking characteristic of AIDS (69–71), is also a feature of CD11c/Nef Tg CD11b^+^ Gr1^+^ cells. Finally, almost all studies of BM histology in a large number of AIDS patients have reported frequent myelodysplasia, hyperplasia, and accumulation of cells of the immature granulocytic and eosinophil lineages (71–79), similar to that found in BM of patients with myelodysplasic syndrome (MDS), to which it was compared (71, 75). This BM phenotype of human AIDS appears to show some similarities with the one described here in Nef Tg mice. Consistent with this, decreased numbers of erythroid colonies (BFU-E) were documented in both Nef Tg models, as in HIV (80–82) or SIV (83, 84) infection. Intriguingly, however, the apparent and well-documented increased number of immature myeloid precursors observed microscopically in BM of HIV- or SIV-infected humans or animals is not reflected in colony assays. Rather, decreased numbers of human (80–82, 85) or simian (83, 84) myeloid progenitor colonies were measured in these *in vitro* assays. This apparent paradox is not understood and may reflect their poor survival *ex vivo* compared to those from Tg mice.

Together, our present data indicate that important phenotypes of human AIDS (BM granulocytic hyperplasia, decreased erythroid precursors, and accumulation of MDSC) can be reproduced in Nef Tg mice and are associated, in these mice, with a number of other characteristics typically found in human AIDS. This suggests that these mouse models, despite their limitations, are helpful in highlighting the critical role of Nef in the development of some AIDS phenotypes and for understanding at least some aspects of myeloid cell biology in human AIDS.

## MATERIALS AND METHODS

### Mice.

C3H/HeNHsd mice were from Harlan and CBA/J, C57BL/6J, BALB/cJ, and FVB/NJ mice (used for the experiment shown in Fig. 6C) from The Jackson Laboratory. The Pep3b (CD45.1) C3H mice have been described (34). CD11c/Nef (CD11c/HIV-Nef) (17), CD4C/Nef (CD4C/HIV^MutG^) (14), CD4C/HIV^MutA^ (14) (coding for Rev, Env, and Nef), CD4C/HIV^MutH^ (14) (coding for all HIV-1 genes except *nef*), CD4C/CD4 (28) Tg and gene-deficient Hck (86), Lyn (87), Src (88), and iNOS (89) mutant mice were previously described. All Tg and knockout (KO) mouse strains were bred with C3H/HeNHsd mice as heterozygous for >6 generations before being studied. Animals were kept in specific-pathogen-free (SPF) rooms. Animal studies followed guidelines set by the Canadian Council on Animal Care and were approved by the Institutional Animal Care Committee. For IL-17 inhibition, CD11c/Nef Tg mice were inoculated with anti-IL-17A Ab (MAB421-500; R&D Systems) or control rat IgG2a (MAB006) intraperitoneally (i.p.), 100 μg/mouse in 0.4 ml phosphate-buffered saline, at day 0, 3, and 6 and sacrificed at day 7.

### Ab and reagents.

All antibodies for flow cytometry were purchased from Cedarlane or BD Biosciences/Pharmingen. Fluorescein isothiocyanate (FITC)-coupled anti-mouse Gr1, CD45.2, I-A^K^ (class II), and Ly6G, phycoerythrin (PE)-coupled anti-mouse Gr1, CD80, CD86, F4/80, H2^K^ (class I), CD45.1, CD62L, and CD71, PE-Cy7-conjugated anti-mouse Ly6C and CD11b, peridinin chlorophyll protein (PerCP)-Cy5.5-conjugated CD45.2 or APC-conjugated CD11c and APC-H7 CD45.1, and biotin-conjugated anti-mouse Ly6C, CD31, CD43, CD124 (with streptavidin-APC or PerCP-Cy5.5) were used to characterize cell surface expression using their appropriate isotypic controls (rat IgG2a, rat IgG2b, and Armenian hamster IgG1). Biotin-coupled anti-mouse CD4, CD8, TCRγδ, DX5, Gr1, CD11b, Ter119, CD19, B220 with streptavidin-APC, or PerCP-Cy5.5 was used for lineage staining and Sca1-PE-Cy7, c-kit-APC-H7, CD34-PE, and CD16/32-FITC for progenitor characterization. APC-conjugated CD4 and PE-Cy7 or PE-CD8 and TCRαβ-FITC were used for T cell characterization. Rabbit anti-pSHIP (Y1020) (Stem Cell Technologies) and goat anti-rabbit Alexa 633 were used for intracellular staining. Mouse anti-human CD4 (4B12) (Thermo Fisher Scientific) and rabbit anti-pStat-5 (Y694) (Cell Signaling) were used for immunofluorescence with chicken anti-mouse Alexa 594 and chicken anti-rabbit Alexa 594, respectively. 7-Aminoactinomycin D (7-AAD) (eBioscience or Cedarlane), annexin V-FITC (BD), anti-CD28, and anti-CD3ɛ (145-2C11) Ab (for T cell stimulation) were bought from Cedarlane, LPS was from Sigma, and anti-biotin MicroBeads for magnetic cell sorting (MACS) were from Miltenyi Biotec.

Antibodies used for immunoblotting were anti-pY (4G10), anti-Hck mAb (Santa Cruz Biotechnology), anti p-Src pY394 (Cell Signaling Technology), anti-S100-8/9, anti-arginase, anti-VEGF-R2, anti-GAPDH (6C5) monoclonal Ab (mAb) (Abcam), anti-actin (Sigma), goat anti-mouse IRDye 800 (LI-COR Biosciences), and anti-mouse IgG (H + L) Alexa 680 (Cedarlane). Anti-bromodeoxyuridine (BrdU)-FITC flow kit was purchased from BD Pharmingen.

### FACS analysis and immunofluorescence.

Flow cytometry was performed as previously published (16, 34). Acquisition was performed on Calibur, BD-LSR (BD Biosciences), or Cyan. Data were analyzed with the CellQuest Pro (BD Biosciences), Summit V4-3, or FlowJo software. Cell sorting was performed on a Mo-Flo cell sorter (Cytomation, Inc.). Immunofluorescence was performed on cytospotted cells, as described previously (16).

### *In vitro* apoptosis of CD11b^+^ Gr1^+^ cells.

Cells (1 × 10^5^ per well) were treated with LPS (1 μg/ml) for 24 h in 6-well plates in 2 ml Iscove complete medium. Apoptosis was estimated by staining with 7-AAD (20 μM) and annexin V-FITC, followed by FACS analysis, as previously described (34).

### ELISA for G-CSF and IL-17 detection.

Reagents to detect G-CSF (number 900-M103) or IL-17 (number 900-M392) by enzyme-linked immunosorbent assay (ELISA) were from Peprotech, Inc., and the assay was run as recommended by the supplier.

### Purification of CD4^+^ and CD8^+^ T cells.

Peripheral (axillary, inguinal, cervical, and brachial) lymph nodes (LN) were collected, and single-cell suspensions were stained with biotin-conjugated anti-mouse CD19, B220, DX5, TcRγδ, Ter119, CD11c, CD11b, and Gr1 for 10 min, followed by incubation with anti-biotin-microbeads for 15 min. T cells were purified by negative selection with an autoMACS system. In some experiments, CD4^+^ T cells were purified using a mouse CD4^+^T cell enrichment kit and EasySep immunomagnetic cell separation (without columns), from StemCell Technologies. The purity of T cells was 95 to 97%, as detected by FACS analysis.

### Purification of suppressor CD11b^+^ Gr1^+^ cells.

CD11b^+^ Gr1^+^ cells were purified with biotin-conjugated anti-Gr1 antibody, followed by MACS anti-biotin-microbeads or cell sorting after staining with anti-CD11b, anti-CD11c, and anti-Gr1 mAb and gating on CD11c-negative cells and CD11b^+^ Gr1^low^ or Gr1^hi^ cells.

### *In vitro* cell proliferation by CFSC fluorescent dye labeling.

CFSE labeling was performed as described previously (90) with a Vybrant CFDA-SE cell tracer kit (Molecular Probe).

### *In vitro* suppression of T cell proliferation by Gr1^+^ cells.

Purified T cells, stained with CFSE, were stimulated in anti-CD3- and anti-CD28 (3 μg/ml)-coated 96-well plates (10^5^ cells/well). Stimulation of AD10 CD4^+^ T cells was done with BM-differentiated DC and Pcc (100 μg/ml) at a ratio of 1:1. Purified CD11b^+^ Gr1^+^ cells (10^5^ well) were added at the same time as T cells (1:1 ratio) in complete medium (250 μl). After 3 days, T cells were analyzed by flow cytometry.

### *In vivo* suppression of T cell proliferation by CD11b^+^ Gr1^+^ cells.

CD4^+^ T cells from donor AD10 TcR mice were purified by negative selection with lineage biotin-conjugated antibodies (mouse CD4^+^ T cell enrichment kit; from Stem Cell Technologies) and stained with 1 μM CFSE for 15 min, washed in RPMI, and injected (3 × 10^6^ to 4 × 10^6^ cells per mouse) intravenously (i.v.) into B10BR female mice. Two days later, 3 × 10^6^ to 4 × 10^6^ CD11b^+^ Gr1^+int/low^ cells purified from spleen of C3H Nef Tg or non-Tg mice, as described above, were injected i.v. Within 1 to 2 h after the latter injection, an equal number of Pcc-loaded DC were injected subcutaneously into recipient mice to stimulate donor AD10 CD4^+^ T cells. The latter DC were prepared from BM maturated with GM-CSF and IL-4 and activated with LPS, as described previously (16), and further incubated with Pcc for 2 h (250 μg/ml). Recipient mice were sacrificed after 3 to 4 days, and proliferation of their T cells was analyzed by FACS.

### BrdU incorporation and analysis.

The BrdU incorporation procedure was described previously (90).

### Colony assay of myeloid progenitors.

Total suspended cells from blood (2 × 10^5^), spleen (5 × 10^4^), and BM (1 × 10^4^) or purified LSK, GMP, and CMP myeloid progenitors (1 × 10^3^) in Iscove medium, 2% FBSI (fetal bovine serum inactivated), 100 U/ml penicillin, and 100 μg/ml streptomycin were used for these assays. Myeloid progenitors were purified by cell sorting using the strategy illustrated in Fig. 4A. Cells were plated in 35-mm dishes at a 1:10 ratio with methylcellulose in the presence of EPO and cytokines or EPO alone (MethoCult GF-M3434 or M3334 [StemCell Technologies]). Cells were cultured at 37°C, 5% CO_2_, and erythroid or myeloid colonies were counted after 5 and 8 to 10 days of incubation, respectively.

### FL cell transplantation.

The FL procedure was previously described (34).

### Western blot analysis and IVKA.

Immunoblot analysis was performed according to a modified procedure reported previously (90). Proteins were visualized with Alexa 680-coupled secondary Ab on the Odyssey imaging system (Li-Cor).

### RT-PCR for HIV-1 Nef.

The reverse transcription-PCR (RT-PCR) assays for quantitation of spliced transgene-specific HIV RNA was performed as described previously (90), using forward primer 377 (5′GCACGGCAAGAGGCGAGGG) at nucleotide 715 of HIV-NL4-3 and reverse primer 1494 (CTAATCGAATGGATCTGTCTCTG) at nucleotide 8460. For quantitative RT-PCR, the same HIV primers were used along with primers for S16 internal standard (forward primer 2432, 5′AGGAGCGATTTGCTGGTGTGG; and reverse primer 2433, 5′GCTACCAGGGCCTTTGAGATG). The reaction mixture contained 5 μl SYBR select master mix (Life Technologies), 0.5 μl of each primer, at 10 pmol/liter and 2 μl template DNA (cDNA) in 2.0 μl RNase-free H_2_O. Amplification was for 40 cycles (15 s at 95°C and 1 min at 60°C) with an AB Applied Biosystems ViiA7 instrument (Life Technologies).

### Statistical analysis.

Statistical analysis was performed in most cases using the unpaired two-tailed Student’s *t* test. In cases of comparison of ratios, a one-sample *t* test was used. One or two-way analysis of variance (ANOVA) with Bonferroni’s correction, where appropriate, was used for comparison of multiple samples. Spearman’s test was used to evaluate correlation of IL-17/G-CSF and G-CSF/CD11b^+^ Gr1^+^ cells.
